# Assessment of immune cell profiles among post-menopausal women in the Women’s Health Initiative using DNA methylation-based methods

**DOI:** 10.1186/s13148-023-01488-8

**Published:** 2023-04-28

**Authors:** Emily Nissen, Alexander Reiner, Simin Liu, Robert B. Wallace, Annette M. Molinaro, Lucas A. Salas, Brock C. Christensen, John K. Wiencke, Devin C. Koestler, Karl T. Kelsey

**Affiliations:** 1 Department of Biostatistics and Data Science, University of Kansas Medical Center, Kansas City, KS USA; 2 Division of Public Health Science, Fred Hutchinson Cancer Center, Seattle, WA USA; 3 Departments of Epidemiology, Medicine, and Surgery, Brown University, Providence, RI USA; 4 Departments of Epidemiology and Internal Medicine, School of Public Health, University of Iowa, Iowa City, IA USA; 5 Department of Neurological Surgery, University of California San Francisco, San Francisco, CA USA; 6 Department of Epidemiology, Geisel School of Medicine, Dartmouth College, Lebanon, NH USA; 7 Department of Molecular and Systems Biology, Geisel School of Medicine, Dartmouth College, Lebanon, NH USA; 8 Department of Community and Family Medicine, Geisel School of Medicine, Dartmouth College, Lebanon, NH USA; 9 UCSF Weill Institute for Neurosciences, University of California San Francisco, San Francisco, CA USA; 10 Departments of Epidemiology and Pathology and Laboratory Medicine, Brown University, 70 Ship St, Providence, RI 02903 USA

**Keywords:** Population, WHI, Immune cell reference limits, Reference values, Immune cell phenotyping, Methylation cytometry, Immunomethylomics, DNA methylation, Deconvolution

## Abstract

**Background:**

Over the past decade, DNA methylation (DNAm)-based deconvolution methods that leverage cell-specific DNAm markers of immune cell types have been developed to provide accurate estimates of the proportions of leukocytes in peripheral blood. Immune cell phenotyping using DNAm markers, termed immunomethylomics or methylation cytometry, offers a solution for determining the body’s immune cell landscape that does not require fresh blood and is scalable to large sample sizes. Despite significant advances in DNAm-based deconvolution, references at the population level are needed for clinical and research interpretation of these additional immune layers. Here we aim to provide some references for immune populations in a group of multi-ethnic post-menopausal American women.

**Results:**

We applied DNAm-based deconvolution to a large sample of post-menopausal women enrolled in the Women’s Health Initiative (baseline, *N* = 58) or the ancillary Long Life Study (WHI-LLS, *N* = 1237) to determine the reference ranges of 58 immune parameters, including proportions and absolute counts for 19 leukocyte subsets and 20 derived cell ratios. Participants were 50–94 years old at the time of blood draw, and* N* = 898 (69.3%) self-identified as White. Using linear regression models, we observed significant associations between age at blood draw and absolute counts and proportions of naïve B, memory CD4+, naïve CD4+, naïve CD8+, memory CD8+ memory, neutrophils, and natural killer cells. We also assessed the same immune profiles in a subset of paired longitudinal samples collected 14–18 years apart across *N* = 52 participants. Our results demonstrate high inter-individual variability in rates of change of leukocyte subsets over this time. And, when conducting paired t tests to test the difference in counts and proportions between the baseline visit and LLS visit, there were significant changes in naïve B, memory CD4+, naïve CD4+, naïve CD8+, memory CD8+ cells and neutrophils, similar to the results seen when analyzing the association with age in the entire cohort.

**Conclusions:**

Here, we show that derived cell counts largely reflect the immune profile associated with proportions and that these novel methods replicate the known immune profiles associated with age. Further, we demonstrate the value this methylation cytometry approach can add as a potential application in epidemiological studies.

**Supplementary Information:**

The online version contains supplementary material available at 10.1186/s13148-023-01488-8.

## Background

Peripheral blood immune cell counts are the entry point for interrogation of the immune system either in response to an environmental insult or therapeutic intervention or in relation to disease states. However, it is important to have well-established reference values for cell types in healthy populations to interpret immune cell counts in clinically relevant populations. Reference values of various leukocyte immune parameters (i.e., cell-type subsets and cell ratios) are numerous in the literature for specific populations/cohorts of individuals across different age groups, races, ethnicities, nationalities, and sexes [1–19].

Some reference values are reported using a complete blood count (CBC), including differential assessed using hematology analyzers, which returns data on both red blood cells and white blood cells (WBC), enumerating five WBC subtypes (neutrophils, eosinophils, basophils, monocytes, and lymphocytes). For example, Cheng et al. [18] used NHANESIII data to develop reference values based on CBC and reported that the granulocyte fraction appears to increase with age, with the lymphocyte fraction showing an inverse association with age. Cheng et al. [18] also reported race-related differences, such as overall WBC count lower in both non-Hispanic black males and females, compared to non-Hispanic white and Mexican American males and females. This trend is a commonly accepted physiologic norm, driven by the inheritance of the Duffy antigen variant [20]. Mononuclear and lymphocyte percentages were increased, and granulocyte percentage was decreased in non-Hispanic African Americans compared to non-Hispanic whites and Mexican Americans. These findings were largely replicated by Coates et al. [7], which included CBC data collected on 7,157 healthy volunteers in the UK. While informative, CBC does not differentiate lymphocyte lineage subtypes, requiring flow cytometry approaches for some specific clinical applications. For instance, CD4+ counts are important in HIV monitoring [21, 22], CD4+ and CD8+ counts have been associated with COVID-19 severity and progression [23, 24] and natural killer cells have been implicated in various autoimmune diseases [25].

Numerous studies have assessed reference values for leukocyte percentages and leukocyte counts using flow cytometry ([1–17, 19] as above). Among the largest and most recent was that of Thyagarajan et al. [1], who studied isolated peripheral blood mononuclear cells (PBMCs) in 8848 participants in the Health and Retirement Study. This population is roughly representative of the US population over 55 years of age. They found total T cells and CD4+ cells declined markedly with age, while CD8+ cells declined with age, though to a lesser degree than CD4+ cells. These findings are independent of cytomegalovirus (CMV) positivity or sex. CD4+ and CD8+ naïve cells were observed to be strongly inversely related to age, and these findings are also independent of CMV positivity. CMV positivity was associated with increases in total T cells and CD8+ cells. Of course, this approach cannot estimate granulocyte prevalence since these cells are lost in the preparation of the sample. One of the few studies to include granulocytes [3] studied 608 Germans and found the number of neutrophils in women to decrease with age, while the relationship between lymphocyte subtypes and age was largely consistent with Thyagarajan et al. [1].

All of the prior studies are beneficial; however, there is opportunity to apply newer research and knowledge to expand upon them. The CBC studies assess only five cell types, and most of the flow cytometry studies are quite small, limiting their power, and they also are conducted primarily on PBMCs where the isolation procedures can introduce additional variability.

DNA methylation (DNAm) deconvolution methods are used to estimate the proportions of leukocyte subsets from peripheral blood. Numerous deconvolution methods have been developed [26–29] with recent efforts focused on expanding the repertoire of immune cell types that can be accurately deconvolved. The application of DNAm deconvolution to estimate immune cell subsets, which we term immunomethylomics or methylation cytometry, is not typically used to establish population-specific reference ranges in large epidemiological studies. In an effort to show the potential use in population research of our recently developed extended DNAm deconvolution library [27], we applied deconvolution methods to Illumina EPIC array DNAm data collected on multi-ethnic participants in a subset of the original Women’s Health Initiative (WHI) participants and a subset of participants in respectively. While Bas has a correlation) in samples that included concurrent CBC, allowing us to estimate both cell counts and cell proportions. This group provides an important population to study factors of immunosenescence and healthy aging, as it is a sample of women ranging in age from 50 to 94 years. With this study, we have implemented highly detailed immune cell phenotyping that includes 58 different immune measures for 1295 individuals to define population-specific reference ranges of DNAm deconvolution estimates and their associations with age and self-reported race.

## Materials/methods

### Study population

The Women’s Health Initiative (WHI) enrolled post-menopausal women nationwide between the ages of 50 and 79 from 1993–1998 to study common causes of morbidity and mortality. There were ~ 160,000 enrolled in one of the clinical trials or an observational study [30]. The Long Life Study (LLS) is an ancillary study (WHI W64) that occurred from 2012 to 2014, about 12–14 years after initial enrollment in WHI. There were 7875 women who completed a one-time in-person visit in which a blood draw was taken, along with clinical and functional status assessments [31]. For a fraction of this LLS group (*N* = ~ 1300), blood samples were used for measuring DNA methylation (DNAm) using the Illumina EPIC array. For some women, blood samples from the original baseline screening for participation in the WHI also had measured DNAm on the EPIC array (*N* = 58). After quality control, 37 samples were removed, yielding a total of 1237 samples from the LLS visit and 58 from the baseline visit. Of these baseline samples, 52 had a matching sample at the LLS visit, meaning there were 104 paired samples and 1191 unique samples. All covariate data was downloaded from the Women’s Health Initiative website (https://www.whi.org/datasets).

### DNA methylation quantification

DNA was extracted from whole blood samples using Five Prime (5 Prime, Inc., Gaithersburg, MD) kits and bisulfite-converted. DNAm was then measured using the Illumina Infinium Human MethylationEPIC BeadChip on the Illumina iScan System as per the manufacturer’s protocol (Illumina, Inc., San Diego, CA; https://www.illumina.com/products/by-type/microarray-kits/infinium-methylation-epic.html), which allows for interrogation of over 850,000 methylation sites. For a specific locus, DNAm was quantified as the proportion of the methylated signal to the sum of the methylation and unmethylated signal, commonly known as the beta(β)-value. Beta-values range from 0 (unmethylated) to 1 (methylated).

### Data preprocessing and quality control

DNAm was measured using the Illumina Infinium MethylationEPIC platform; probe intensity data (IDATs) were obtained from the WHI. Data were processed using the *minfi* (v.1.36.0) [32] and *ENmix* (v.1.26.10) [33] Bioconductor packages using R version 4.0.3. Background correction and dye-bias normalization was conducted using Noob [34] via the preprocessNoob function in the *minfi* Bioconductor package. To assess data quality, low-quality data points were defined as an out-of-band detection *P*-value threshold of > 0.05 or a beads per probe threshold of > 3. Low-quality data points were set as missing values. Low-quality samples were then defined as samples with greater than 5% missing values across CpGs, bisulfite intensity below the mean minus 3 standard deviations, or outliers in the beta distribution. Low-quality CpGs were defined as CpGs with greater than 5% missing values across samples. After quality control, 37 low-quality samples and 25,816 low-quality CpGs were removed and excluded from subsequent analyses. Further, probes on sex chromosomes and CpH probes were removed, and the Zhou et al. [35] general masking was used to remove probes, which are cross-reactive, polymorphic, and associated with SNPs. In total, 1295 samples and 729,797 CpGs were retained for downstream analyses.

### Estimation of immune parameters

Leukocyte cell-type proportions were estimated via reference-based DNAm deconvolution using the 12-cell-type reference library, EPIC IDOL-Ext, contained in the *FlowSorted.BloodExtended.EPIC* Bioconductor package [27]. Three probes (cg12810503, cg11415852, cg08911152) that are part of the 12-cell-type reference library were excluded due to poor quality and thus were not used in deconvolution. The 12 cell types in this library include: neutrophils (Neu), eosinophils (Eos), basophils (Bas), monocytes (Mono), memory B cells (Bmem), naïve B cells (Bnv), naïve CD4+ cells (CD4nv), memory CD4+ cells (CD4mem), naïve CD8+ cells (CD8nv), memory CD8+ cells (CD8mem), T regulatory cells (Treg) and natural killer cells (NK). If a deconvolution estimate fell below the limit of detection for a cell type for a sample, the estimate was replaced with the limit of detection value [36] (Additional file 1: Table S1). Then, cell-type proportion estimates were multiplied by the respective total white blood cell (WBC) count provided in the WHI covariate data to obtain estimates of absolute counts (in cells per microliter). Counts and proportions for various cell types, including lymphocytes (Lymph), total T cell (Tcell), CD4+ T cells (CD4), CD8+ T cells (CD8), B cells (Bcell), myeloid (Mye), and granulocyte (Gran), were derived by summing their individual components (e.g., Bcell = Bmem + Bnv). We also derived values for multiple cell ratios, giving 58 immune parameters estimated in total (Additional file 2: Fig. S1). There was one WHI-LLS sample for which the WBC count was not available.

### Statistical analysis

Absolute cell counts and cell-type proportions were stratified by common demographic variables used in clinical settings and epidemiological studies: age and self-reported race. Age was grouped into deciles starting at age 50, and the self-reported race was stratified by White (*N* = 898) and Black (*N* = 370). Those who identified as other race (*N* = 17), American Indian/Alaska Native (*N* = 1), or those who did not report their race (*N* = 9) were excluded from race-specific stratifications due to small sample sizes. Such individuals were, however, included in the overall tables that look at the entire population (*N* = 1294 for cell counts and *N* = 1295 for cell proportions and cell ratios). Immune cell absolute counts (cells/µl) and cell-type proportions (as the percent of the total WBC) were summarized by the mean, 95% confidence interval of the mean, median, and 2.5–97.5 percentile. To test the effect of age on immune cell parameters, linear regression models were fitted, modeling either the proportion (%) or absolute counts (cells/µl) of an immune cell type as the dependent variable and age (continuous) as the independent variable. Models were also adjusted for Duffy antigen genotype, as this is well known to be associated with neutropenia [20], and thus could have an effect on other cell types. Linear regression was also used to test the effect of age on cell-type ratios (e.g., NLR) in the same way. Models were fitted separately for self-identified White women (*N* = 895) and self-identified Black women (*N* = 367) who had the Duffy antigen genotype available (Additional file 1: Table S8), totaling 88 regression models all together. For each model, interactions between the independent variables were assessed for statistical significance. Paired t tests were used to test differences for longitudinal samples. The comparison of the rate of change of longitudinal samples by age was analyzed using simple linear regression. A *p*-value < 0.05 was considered significant for all tests.

### Software

R version 4.1.3 was used for all statistical analyses.

## Results

### Demographics

Table 1 summarizes the characteristics of the population in this study (*N* = 1295). The mean age at blood draw is 78.96 (SD ± 7.5) and ranges from 50 to 94 years of age; this includes women from both the Women’s Health Initiative (WHI) baseline enrollment (*N* = 58) and the WHI Long Life Study (LLS) (*N* = 1237). Most of the population are White women (69.3%), 28.6% are Black women, and 7.1% of the participants report Spanish, Hispanic, or Latino ethnicity. The Duffy Antigen Receptor for Chemokines (DARC) null genotype was found to be present in 18.8% of this population. The mean BMI is 28.43 (SD ± 5.9), with 29.9% of women categorized as normal weight, 35.8% as overweight, and 33.4% as obese. Only 2.7% of the women were current smokers at the time of blood draw, and over half (53.6%) of this population indicated that they had never been smokers. The vast majority of this population reported their general health as good, very good, or excellent (79.7%), while 10.6% reported it as fair or poor.

**Table 1:** Demographics of WHI baseline and LLS individuals whose DNAm samples were used in this study

Variable name	Valueƚ
*Samples*	
Paired	104 (8)
Unique	1191 (92)
Total	1295
*Visit*	
Baseline	58 (4.5)
LLS	1237 (95.5)
Age	78.96 (7.5)
*Age group*	
50–59	21 (1.6)
60–69	122 (9.4)
70–79	454 (35.1)
80–89	632 (48.8)
90+	66 (5.1)
Race*	
White	898 (69.3)
Black	370 (28.6)
Other^ǂ^	17 (1.3)
American Indian/Alaska Native	1 (0.08)
Missing	9 (0.7)
Ethnicity*	
Spanish/Hispanic/Latino	92 (7.1)
*Duffy Antigen Receptor for Chemokines genotype*	
FY−/−(DARC CC)	243 (18.8)
FY−/+(DARC CT)	128 (9.9)
FY+/+(DARC TT)	918 (70.9)
Missing	6 (0.5)
BMI (kg/m^2^)	28.43 (5.9)
*BMI categories*	
Underweight (< 18.5)	13 (1.0)
Normal (18.5–24.9)	381 (29.9)
Overweight (25.0–29.9)	457 (35.8)
Obesity I (30.0–34.9)	269 (21.1)
Obesity II (35.0–39.9)	99 (7.8)
Extreme obesity (> = 40)	57 (4.5)
General health**	
Excellent	84 (6.5)
Very good	444 (34.3)
Good	504 (38.9)
Fair	129 (9.9)
Poor	9 (0.7)
Missing	125 (9.7)
*Smoking*	
Never smoker	694 (53.6)
Ever smoker	494 (38.1)
Current smoker	35 (2.7)
Missing	72 (5.6)
*CBC availability*	
Total WBC count	1294 (99.9)
Differential	1237 (95.5)

*Self-identified race and ethnicity

**Self-reported general health

ƚValues are *N*(%), except for age and BMI which is mean(s.d.)

ǂ”Other” was a category participants could identify with, and not a grouping of races

### Estimates from DNAm deconvolution are comparable to estimates from CBC differentials

**Fig. 1:**
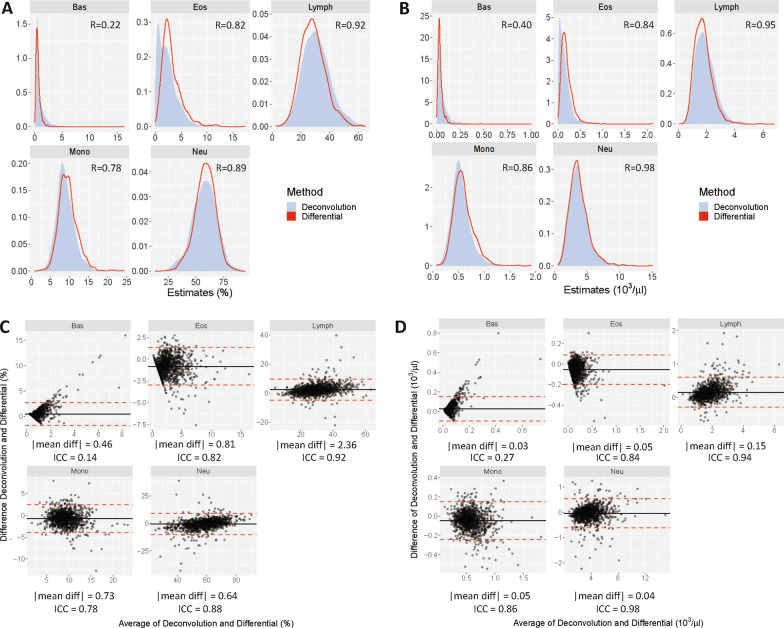
Validity of DNA methylation deconvolution estimates. Density plots, correlation coefficients, and Bland–Altman plots comparing the estimates from deconvolution to the estimates from a CBC differential. **A**, **B** Density plot of estimates for proportions (**A**), represented as percentages, and absolute counts (**B**), represented as 10^3^/µl. The red line represents the distribution of estimates from the differential, and the light blue curve represents the distribution of estimates from using deconvolution. The value for *R* represents the Pearson correlation coefficient of the estimates for the two methods. **C**, **D** Bland–Altman plots to visualize the agreement between the two methods using the estimates for proportions (**C**) and absolute counts (**D**). The x-axis is the average of the estimate from the differential and deconvolution. The y-axis is the difference in the estimate from the deconvolution and differential. The horizontal black line is located at the average difference of the estimates, and the two dashed red lines are at the 95% confidence interval for the average difference. Each point is a sample. (ICC = intraclass correlation coefficient)

We first estimated the proportion of neutrophils (Neu), eosinophils (Eos), basophils (Bas), monocytes (Mono), memory B cells (Bmem), naïve B cells (Bnv), naïve CD4+ cells (CD4nv), memory CD4+ memory cells (CD4mem), naïve CD8+ cells (CD8nv), memory CD8+ cells (CD8mem), T regulatory cells (Treg) and natural killer cells (NK) for each sample via reference-based DNAm deconvolution via the 12 leukocyte subtype reference library described in Salas et al. [27]. Using total white blood cell (WBC) counts for each sample provided by the WHI, we also derived estimates of the absolute count of each cell type as cells per microliter (Additional file 2: Fig. S1). These estimates of proportions and counts were compared to the respective values for Bas, Eos, Mono, Neu, and Lymph obtained from a CBC differential, which was available for most of the samples (Table 1, *N* = 1237). The Pearson correlation between deconvolution estimates and CBC differential estimates are large for Eos (*R* = 0.82, *R* = 0.84), Mono (*R* = 0.78, *R* = 0.86), Neu (*R* = 0.89, *R* = 0.98), and Lymph (*R* = 0.92, *R* = 0.95) for proportions and absolute counts, respectively. While Bas has a correlation coefficient of 0.40 for absolute counts and 0.22 for proportions (Fig. 1A and B). The agreement between the two methods is fairly high, with a less than 1% absolute mean difference in deconvolution derived proportions and CBC estimated proportions for Bas, Eos, Mono and Neu and a 2.36% absolute mean difference for Lymph (Fig. 1C). When comparing counts derived from deconvolution and counts from the CBC, there is ≤ 50 cells/µl absolute difference for Bas, Eos, Mono, and Neu, and an absolute mean difference of 150 cells/µl for Lymph (Fig. 1D). Additionally, the majority of samples fall within the 95% confidence interval for the average difference between the deconvolution estimates and the CBC differential estimates for all the cell types (Fig. 1C and D). The intraclass correlation coefficient (ICC) was also computed for the two methods. Basophils show poor reliability between the two methods (ICC = 0.14 cell proportions, ICC = 0.27 absolute counts). However, for proportions and counts of Eos (ICC = 0.82, ICC = 0.84), Lymph (ICC = 0.92, ICC = 0.94), Mono (ICC = 0.78, ICC = 0.86), and Neu (ICC = 0.88, ICC = 0.98), there is good to excellent reliability between the two methods.

### Reference ranges established for 58 immune cell parameters

To establish references ranges and characterize immune cell parameters derived using DNAm deconvolution estimates based on common demographic variables, data was grouped by age using deciles, starting from 50–59 years to 90+ years, and self-reported race, either Black or White. The immune cell types and ratios were summarized by their proportion (%) and absolute counts (cells/µl) as the mean and 95% confidence interval, median, and 2.5–97.5 percentile. Table 2 summarizes estimates for the entire population (*N* = 1295 for % or ratios and *N* = 1294 for cells/µl). There is a high correlation between derived proportions and their subsequent derived counts, with increasing variability as the deconvolution estimates increase (Additional file 3: Fig. S2). Additional file 1: Tables S2, S3, and S4 summarize estimates stratified by age and self-reported race. This totals 58 immune cell parameters (Additional file 2: Fig. S1) in which reference ranges are defined for this population of aging, post-menopausal women.

**Table 2:** Summary of immune cell parameters based on DNA methylation deconvolution estimates for the entire cohort

	Mean	(95% CI)	Median	Percentile range (2.5–97.5)
WBC*	6276.028	(6173.04, 6379.015)	6000	(3386.5, 10,680.25)
*Leukocyte subtypes*				
Myeloid**				
Cells/µl	4394.189	(4304.664, 4483.714)	4198.418	(1893.226, 8212.533)
%	69.161	(68.621, 69.701)	69.949	(46.791, 86.387)
Granulocytes**				
Cells/µl	3848.037	(3763.108, 3932.967)	3668.982	(1503.92, 7470.417)
%	60.231	(59.648, 60.815)	60.94	(36.342, 78.904)
Neutrophils				
Cells/µl	3642.355	(3558.256, 3726.454)	3469.95	(1334.334, 7201.745)
%	56.833	(56.207, 57.459)	57.457	(31.576, 76.845)
Basophils				
Cells/µl	65.7	(61.971, 69.43)	41.723	(18.371, 242.819)
%	1.08	(1.018, 1.141)	0.6	(0.478, 3.474)
Eosinophils				
Cells/µl	139.982	(133.382, 146.582)	112.492	(19.425, 444)
%	2.319	(2.219, 2.418)	2	(0.422, 6.539)
Monocytes				
Cells/µl	546.152	(536.271, 556.033)	524.352	(261.548, 961.111)
%	8.929	(8.801, 9.058)	8.66	(4.931, 14.337)
Lymphocytes**				
Cells/µl	1920.642	(1881.476, 1959.808)	1822.981	(842.129, 3620.853)
%	31.465	(30.92, 32.01)	30.507	(14.382, 54.122)
T cells**				
Cells/µl	1358.107	(1327.532, 1388.681)	1280.328	(525.1, 2645.941)
%	22.247	(21.809, 22.685)	21.155	(9.341, 40.643)
CD4+ total**				
Cells/µl	859.971	(838.43, 881.512)	794.503	(296.086, 1799.017)
%	14.139	(13.811, 14.467)	13.283	(5.096, 28.434)
CD4+ memory				
Cells/µl	609.586	(593.698, 625.473)	564.328	(170.535, 1328.026)
%	10.058	(9.81, 10.306)	9.378	(2.796, 21.22)
CD4+ naïve				
Cells/µl	200.559	(190.288, 210.829)	132.151	(34.738, 685.529)
%	3.283	(3.118, 3.447)	2.28	(0.878, 11.362)
T regulatory				
Cells/µl	49.826	(48.874, 50.779)	46.956	(26.777, 87.899)
%	0.798	(0.785, 0.81)	0.78	(0.78, 0.78)
CD8+ total**				
Cells/µl	498.136	(476.832, 519.44)	397.338	(84.81, 1473.431)
%	8.108	(7.793, 8.423)	6.715	(1.559, 22.443)
CD8+ memory				
Cells/µl	418.013	(396.375, 439.651)	306.012	(37.325, 1385.639)
%	6.803	(6.478, 7.128)	5.278	(0.801, 21.569)
CD8+ naïve				
Cells/µl	80.123	(76.878, 83.368)	58.101	(28.146, 246.615)
%	1.305	(1.253, 1.357)	0.758	(0.758, 4.127)
B cells**				
Cells/µl	227.216	(218.517, 235.914)	189.396	(50.299, 640.404)
%	3.755	(3.617, 3.893)	3.183	(0.811, 9.76)
B memory				
Cells/µl	86.814	(81.799, 91.829)	67.363	(16.061, 255.53)
%	1.412	(1.335, 1.489)	1.172	(0.328, 4.097)
B naïve				
Cells/µl	140.402	(133.852, 146.952)	106.589	(23.055, 503.33)
%	2.343	(2.234, 2.452)	1.834	(0.483, 7.108)
Natural killer				
Cells/µl	335.32	(326.172, 344.467)	308.259	(94.861, 761.925)
%	5.463	(5.331, 5.596)	5.144	(1.587, 11.042)
*Cell ratios*				
Neutrophil/lymphocyte	2.162	(2.087, 2.236)	1.879	(0.597, 5.281)
Lymphocyte/monocyte	3.699	(3.625, 3.774)	3.502	(1.635, 6.829)
Eosinophil/lymphocyte	0.077	(0.073, 0.081)	0.063	(0.012, 0.253)
Basophil/lymphocyte	0.038	(0.035, 0.04)	0.024	(0.01, 0.134)
Eosinophil/neutrophil	0.046	(0.044, 0.048)	0.035	(0.006, 0.155)
(Eosinophil + Basophil)/neutrophil	0.067	(0.064, 0.071)	0.053	(0.012, 0.209)
Neutrophil/monocyte	7.126	(6.88, 7.372)	6.482	(2.855, 14.15)
CD4/CD8	3.012	(2.857, 3.166)	2.063	(0.35, 11.097)
CD4nv/CD8nv	3.014	(2.854, 3.174)	1.832	(0.419, 11.073)
CD4mem/CD8mem	3.806	(3.563, 4.049)	1.903	(0.275, 15.93)
CD4nv/CD4	0.218	(0.211, 0.226)	0.189	(0.047, 0.518)
CD4nv/CD4mem	0.365	(0.347, 0.383)	0.256	(0.052, 1.252)
CD8nv/CD8	0.266	(0.254, 0.279)	0.173	(0.034, 0.769)
CD8nv/CD8mem	0.61	(0.561, 0.659)	0.209	(0.035, 3.331)
Treg/CD4	0.069	(0.066, 0.071)	0.059	(0.027, 0.165)
Bnv/B	0.591	(0.579, 0.602)	0.607	(0.177, 0.919)
Bnv/Bmem	2.628	(2.44, 2.816)	1.544	(0.215, 11.359)
CD8/B	3.107	(2.918, 3.296)	1.999	(0.357, 12.643)
(CD8+ NK)/Mono	1.604	(1.555, 1.653)	1.427	(0.498, 3.788)
CD8mem/Treg	8.684	(8.269, 9.1)	6.752	(1.027, 27.648)

*WBC = White blood cell count, from WHI data. All other cell count data is from multiplying cell deconvolution proportion estimates by WBC

**Derived cell counts: Lymph = CD4-total + CD8-total + B-total + NK + Treg; Tcell = CD4-total + CD8-total; CD4-total = CD4mem + CD4nv + Treg; CD8-total = CD8mem + CD8nv; B-total = Bmem + Bnv; Myeloid = Gran + Mono; Gran = Neu + Eos + Bas

### Immune cell parameters are associated with age and race regardless of Duffy antigen genotype

**Fig. 2:**
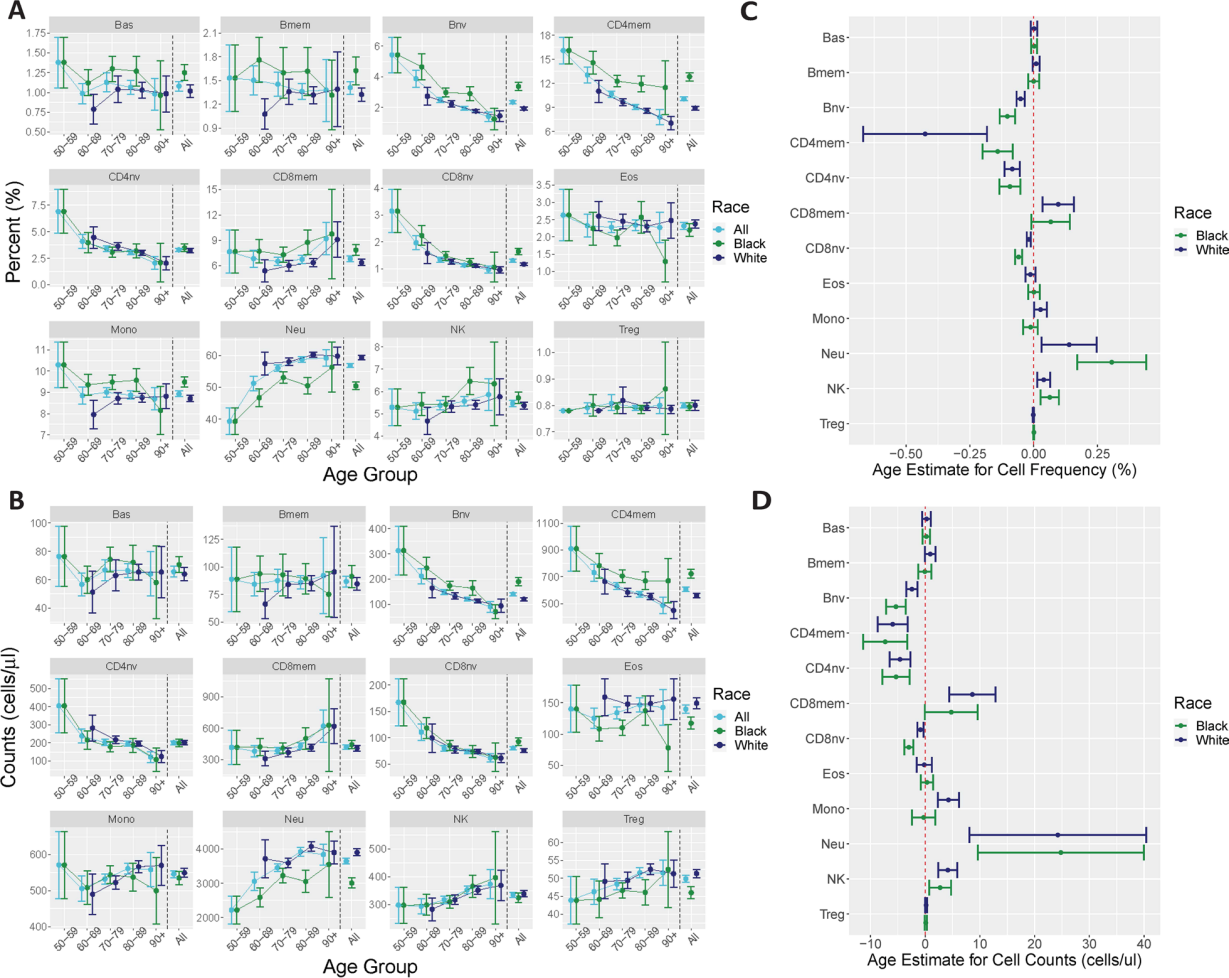
Associations of immune cell profiles with age and race. **A**, **B** Line plots showing the proportion estimates as percentages from deconvolution (**A**) and absolute counts (**B**) in cells/µl, stratified by age and self-reported race. Data are represented by the mean values and 95% CIs. The y-axis is the percent value or the cells/µl value. The x-axis is age group, and self-identified race is indicated by color (light blue = all participants (*N* = 1295), dark-green = Black women (*N* = 367), dark-purple = White women (*N* = 895)). **C**, **D** Regression coefficients for age, after adjusting for Duffy antigen genotype, for proportions (%/year) (**C**) and absolute counts (cells/µl/year) (**D**) stratified by self-reported race. Data are represented as the regression coefficient for age and the 95% CI

To test the effect of age on immune cell parameters, linear regression models were fitted modeling proportions and counts of immune cell parameters by age, controlling for Duffy antigen genotype. Models were stratified by self-reported race. For both Black and White women, there were significant associations with age for proportions and counts of Bnv, CD4mem, CD4nv, CD8nv, Neu and NK cells (Fig. 2, Additional file 1: Table S5). CD4mem and CD4nv cell proportions and counts decreased as age increased at similar rates between Black women and White women, and Neu and NK cells increased at similar rates (Fig. 2, Additional file 1: Table S5). There was a significant interaction between age and Duffy antigen genotype for CD4mem fraction only among White women, along with significant interaction for NK cells, but only among Black women (Additional file 1: Table S5). Compared to White women, Black women had an increased rate of cell decline as age increased for Bnv (− 5.351 cells/year vs. − 2.428 cells/year and − 0.103%/year vs. − 0.051%/year) and CD8nv (− 2.986 cells/year vs. − 0.860 cells/year and − 0.059%/year vs. − 0.019%/year). Only White women saw a significant increase in CD8mem and Mono proportions and counts as age increased (Fig. 2, Additional file 1: Table S5).

**Fig. 3:**
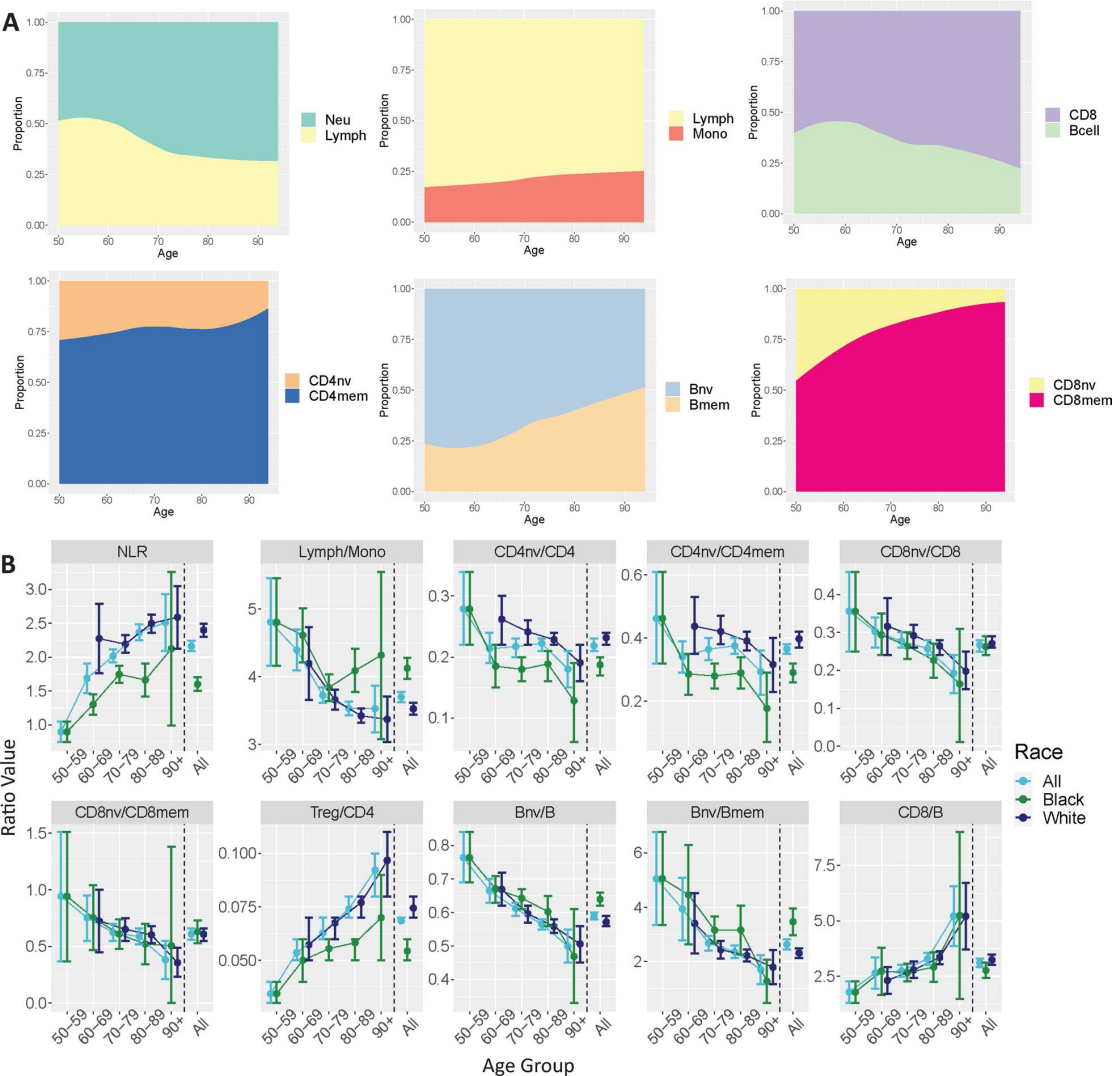
Changes in cell ratios across age and race. **A** Area plots showing the average and smoothed cell proportion per compartment across age. **B** Line plots showing the cell ratios stratified by age and self-reported race. Data are represented by the mean values and 95% CIs. The y-axis is the ratio value. The x-axis is age group, and self-reported race is indicated by color (light blue = all participants (*N* = 1295), dark green = Black women (*N* = 367), dark purple = White women (*N* = 895))

The Neu/Lymph (NLR), Lymph/Mono, CD4nv/CD4, CD4nv/CD4mem, CD8nv/CD8, CD8nv/CD8mem, Treg/CD4, Bnv/B, Bnv/Bmem, and CD8/Bcell ratios all had significant linear associations with age in both Black women and White women, and even upon adjustment for Duffy antigen genotype (Fig. 3, Additional file 1: Table S5). The NLR, Treg/CD4 and CD8/B ratios increased as age increased, while the others decreased as age increased (Fig. 3, Additional file 1: Table S5). The Eos/Neu, CD4/CD8, CD4nv/CD8nv, and CD4mem/CD8mem significantly decreased as age increased in White women, while the CD8mem/Treg and (CD8+ NK)/Mono significantly increased in White women (Additional file 1: Table S5, Additional file 4: Fig. S3). There were no significant associations between these ratios for Black women. The Bas/Lymph and Neu/Mono ratios significantly increased as age increased in Black women only (Additional file 1: Table S5, Additional file 4: Fig. S3).

### Longitudinal samples show similar associations with age with high variability between individuals

**Fig. 4:**
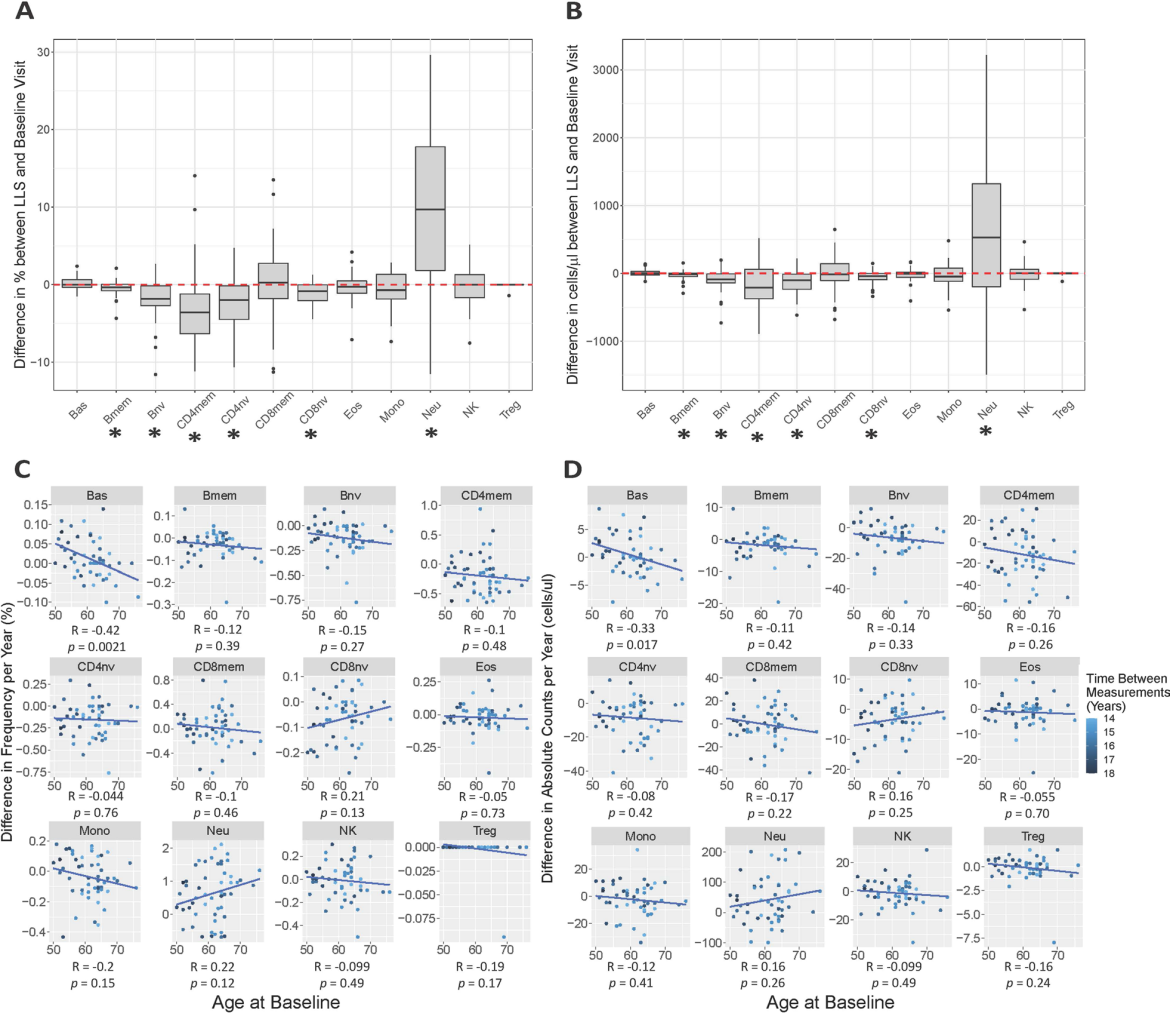
Immune cell profiles in paired, longitudinal samples. **A**, **B** Box plots showing the change in (**A**) proportion estimates and (**B**) absolute counts for paired samples. Change was calculated as the difference from the baseline visit to the LLS visit for a pair of samples (LLS estimate—baseline estimate). Red dashed line is at a change of 0. Starred cell types indicate *p*-value < 0.05 when conducting a paired t-test. **C**, **D** Scatter plots of the rate of change for longitudinal samples by age of the subject. Each point is the difference in proportion per year (**C**) or difference in absolute counts per year (**D**) from the baseline visit to the LLS visit for a pair of samples for a given subject. The points are colored in by the number of years between the pair of samples. Pearson correlation coefficients and *p*-values for associations between rate of change and age at baseline are below each cell type plot

The results of the overall association of immune cell parameters and age were mirrored when examining the 52 individuals with longitudinal blood samples (Fig. 4A, B; Additional file 5: Fig. S4). Of the 104 paired samples, one sample came from the original baseline visit from enrollment in the WHI, and the second sample was from the WHI-LLS visit. There is a range of 14–18 years between two samples of the same individual; most of these samples come from Black women (*N* = 48). Using paired t tests, there are significant decreases from baseline to the LLS visit in cell proportions and counts for Bmem (|Δ|= 0.47% and 28.8 cells/µl, *p* = 0.007 and 0.002), Bnv (|Δ|= 1.86% and 101.3 cells/µl, *p* < 0.0001 and 0.0001), CD4mem (|Δ|= 2.96% and 178.3 cells/µl, *p* < 0.0001 and *p* = 0.0003), CD4nv (|Δ|= 2.35% and 131.3 cells/µl, *p* < 0.0001 and 0.0001), and CD8nv (|Δ|= 1.05% and 54.2 cells/µl, *p* < 0.0001 and *p* = 0.0002). There is a significant increase in Neu proportions (|Δ|= 9.54%, *p* < 0.0001) and counts (|Δ|= 651.4 cells/µl, *p* = 0.0002). However, NK cells did not show a significant increase in proportion or absolute counts in the longitudinal samples. The rates of change (calculated as the difference in proportion or absolute counts per year) across subjects is highly variable (Fig. 4C, D; Additional file 1: Table S6). For example, Neu range from − 0.69 to +2.11%/year and − 99.51 to +207.68 cells/µl/year. However, using simple linear regression, the association of age and rate of change was not significant for most cell types. The one exception is for Bas (*R* = − 0.42, *p* = 0.0021 for proportions, *R* = − 0.33, *p* = 0.017 for counts, Fig. 4C, D).

### Comparison immune cell counts to those in the literature

We conducted a literature review of studies that include reference ranges derived from flow cytometry methods and CBC differentials for similar populations to this study. Table 3 shows a selected comparison from the full literature review of our estimates of 15 immune cell parameters to 4 other studies with the most similar populations. Additional file 1: Table S7 includes all studies reviewed.

**Table 3:** Comparing immune cell counts to those in the literature (selected version)

Study		WHI (this study)			Provinciali et al. [17]^a^	Thyagarajan et al. [1]^b^	Valiathan et al. [11]^c^		Melzer et al. [3]^d^	
Geographic area		United States			Italy	United States	United States		Germany	
Sex		Women			Women	Women	Women		Women	
Age range		50–94			46–90+	56–86+	70–92		60–79	
Sample size		1294			191	4874	33		195	

Method		DNAm deconvolution			Flow cytometry	Flow cytometry	Flow cytometry		Flow cytometry	
Type		Percentile (2.5–97.5)	Mean	Median	Weighted mean	Weighted mean	Mean	Median	Median	Percentile (2.5–97.5)
Neutrophils	Cells/µl	(1334, 7202)	3642	3470					3122	(1801, 5579)
Eosinophils	Cells/µl	(19, 444)	140	112					101	(30, 383)
Monocytes	Cells/µl	(262, 961)	546	524					230	(108, 422)
Lymphocytes	Cells/µl	(842, 3621)	1921	1823					1624	(677, 2916)
T cells	Cells/µl	(525, 2646)	1358	1280		1434	1424	1351	1098	(369, 2086)
CD4+ total	Cells/µl	(296, 1799)	860	795	710	999	892	867	818	(263, 1707)
CD4+ memory	Cells/µl	(171, 1328)	610	564	324	384				
CD4+ naïve	Cells/µl	(35, 686)	201	132	176	486				
T regulatory	Cells/µl	(27, 88)	50	47					60	(22, 130)
CD8+ total	Cells/µl	(85, 1473)	498	397	410	337	525	453	143	(32, 437)
CD8+ memory	Cells/µl	(37, 1386)	418	306	178	200				
CD8+ naïve	Cells/µl	(28, 247)	80	58	59	73				
B-total	Cells/µl	(50, 640)	227	189			229	164	216	(62, 513)
Natural Killer	Cells/µl	(95, 762)	335	308			339	250	276	(85, 624)
CD4/CD8		(0.35, 11.1)	3.01	2.07			2.4	1.7		

^a^Weighted means were calculated from data in Table 1 of Provinciali et al.

^b^Weighted means were copied from Additional file 1: Table S3 of Thyagarajan et al. and converted to cells/µl; CD4mem and CD8mem were added from their respective TCM + Effector + TEM compartments

^c^Mean and median values were copied from Additional file 1: Table S2 of Valiathan et al.

^d^Median and percentile values were copied from Additional file 1: Table S4 of Melzer et al. and converted to cells/µl; CD4 were taken from T helper (Th) cells, CD8 were taken from cytoxic T (Tc) cell

## Discussion

We applied our novel approach to peripheral blood methylation data in a subset of the WHI to establish reference ranges across 58 immune cell parameters. The deconvolution method used was recently developed by Salas et al. [27] where libraries for this high resolution immune phenotyping were created and validated against the gold standard of flow cytometry. This technique provides the opportunity for estimating proportions and counts for leukocyte subtypes without the need for fresh blood and in a cheaper and more standardized fashion compared to flow cytometry, thus making it scalable to large sample sizes. In these data, we showed deconvolution estimates were similar to complete blood cell count (including differential) data in the WHI, showing it to be comparable to conventional assessment of peripheral blood immune profile in the setting of blood collected as part of a large epidemiologic study. Our results showed a high correlation between the proportions and count data, and the deconvolution estimates were almost all within the 95% confidence limits of the differential estimates from the initial blood draw. Further, the magnitude of difference between the two methods falls within the range seen in other studies [37, 38]. The overall leukocyte count data allows us to convert the relative prevalence to a count, making comparisons to prior published data enumerating immune profiles in blood using flow cytometry. This further validates this method and demonstrates its potential for application in other large epidemiologic studies.

There was little disagreement when we compared our values for immune cell counts to those in the literature derived from flow cytometry methods and CBC differentials (Table 3, Additional file 1: Table S7). There were no studies that established reference ranges for all the immune parameters defined in this study and few studies that share a similar population as compared to the present study, mostly due to the age range. The studies with the most similar populations are Provinciali et al. [17], Thyagarajan et al. [1], and Valiathan et al. [11]; however, they only established reference ranges for various lymphocyte subsets using PBMCs (which may introduce variability in counts). The estimates derived for CD4+ and CD8+ cells and their naïve and memory subsets in Provinciali et al. [17] and Thyagarajan et al. [1] compared to the estimates derived in this study for the WHI cohort are fairly similar (Table 3), except for CD8mem mean absolute counts being much higher in the WHI compared to both Provinciali et al. [17] and Thyagarajan et al. [1] (Table 3; WHI = 418 cells/µl, Provinciali = 178 cells/µl, Thyagarajan = 200 cells/µl). Valiathan et al. [11] had a much smaller sample size (*N* = 33) of women in the same age range compared to the WHI. However, mean and median absolute counts of the total CD4+, CD8+, B, and NK cells were most comparable to those derived in the WHI (Table 3). Finally, Melzer et al. [3] included cell-type estimates of Neu, Eos, Mono, Lymph, total T, CD4+ total, Treg, CD8+ total, total B, and NK cells for a group of women ranging from 60 to 79 years of age, which is a similar age group to about 45% of the WHI cohort. The study's most similar median absolute count estimates were Eos, CD4+, Treg, total B, and NK cells (Table 3). As noted, across all other studies, most cell type estimates were in agreement to those derived in our study (Additional file 1: Table S7). One exception is CD8mem, where, similar to the above studies, we observed much larger absolute counts in the WHI than all other studies; this may be the result of small sample sizes of the other studies, systematic loss of CD8+ cells in PBMC preparation or perhaps chance.

Further evidence of the utility of this approach is found in the data showing that immune profiles derived from deconvolution demonstrate well-known relationships of age with immune subsets. For example, we show the expected diminution of the naïve compartments of CD4+ [1, 2, 17, 19, 39–42] and CD8+ [1, 17, 39–44] lymphocyte lineages, as well as the expected growth of CD8mem [1, 39, 42]. Also, similar to other published studies, we see a decrease in Bnv [44] and an increase in Neu [11] and NK cells [8, 11, 12] with age. However, in contrast to other studies [1, 2, 17, 19, 39, 42], we show a significant decrease in CD4mem with age. We also show that the well-established Duffy antigen variant genotype predicts the expected neutropenia [20] assessed by both percentages and counts.

We have also presented novel reference ranges for numerous additional immune parameters that may find considerable utility in epidemiologic studies going forward. For example, the NLR has been widely used in studies of cancer survival [45–47]. This is now being applied to cancer risk as well as the risk of additional inflammatory-associated diseases (e.g., diabetes [48, 49], cardiovascular disease [50, 51], and stroke [52, 53]). We find many of the novel immune parameters that are generated easily using this method also are associated with age and self-reported race. Our data may be quite valuable for comparison with other epidemiologic studies.

Finally, we made use of methylation data from blood draws repeated over a number of years in the same subject to assess individual changes in immune profile over an average of some 14–18 years. This is very unusual data and shows that changes in immune profiles in elderly women are highly variable. Other studies have reported this high inter-individual variability for similar cell types, including Lin et al. [44], which had paired longitudinal samples 5 years apart, and Alpert et al. [43], which had longitudinal samples collected over 9 years. This may hold important information and thus suggests that larger studies nested in ongoing large cohorts where multiple blood draws have occurred may hold novel findings of changes in immune profiles predicting subsequent types of chronic inflammatory disease.

This study is not without limitations. We only compared our deconvolution estimates within the WHI samples using CBC derived estimates, which lack the granularity to distinguish various lymphocyte subsets. Although we could not validate the deconvolution estimates with flow cytometry on the same samples, reference-based deconvolution using DNAm has been shown to be accurate across numerous studies [26, 27, 29]. We also did not have access to CMV status and thus could not test this association with the immune profile. Although we use self-reported race to describe immune cell profiles, we are limited in interpretation by not addressing other factors that could be relevant (e.g., environmental factors). However, interpretation for why there might be differences between groups is beyond the scope of this work but does provide the opportunity for future studies. Further, our study population included only women aged 50–94 years old at the time of blood draw, thus limiting generalizability to younger women or men.

## Conclusions

We have outlined a method to provide highly detailed immune phenotyping using stored peripheral blood samples and validated this method using complete blood count differential estimates and comparisons to estimates from flow cytometry from other published studies. Although this work was conducted in one particular population, it demonstrates the validity of the methylation cytometry approach and should stimulate additional investigation of immune profiles and chronic disease associations in prospect cohorts.

## Supplementary Information

**Additional file 1: Table S1**. Limit of detection for DNAm deconvolution estimates.** Table S2**: Summary of absolute counts of immune cells stratified by age and race.** Table S3**: Summary of proportions of immune cells stratified by age and race.** Table S4**: Summary of immune cell ratios stratified by age and race.** Table S5**: Results from linear regression analysis.** Table S6**: Rate of change of immune cell parameters in paired longitudinal samples.** Table S7**: Comparing immune cell counts to those found in literature.** Table S8**: Distribution of the Duffy antigen genotype.

**Additional file 2. Fig. S1**: Study design for deriving all 58 immune parameters. (1) First, DNAm deconvolution is performed using the DNAm measured from peripheral blood samples. The proportions of 12 leukocyte subsets are given. (2) Derive other leukocyte subsets by adding respective components together. Also, derive absolute counts of all the subsets by multiplying the proportion by the total WBC. (3) Derive cell ratios. This gives 58 immune parameters in total.

**Additional file 3. Fig. S2**: Correlation between deconvoluted proportions and derived cell counts. Scatter plots showing the correlation between DNAm deconvoluted proportions and their respective derived cell counts for each of the 12 subtypes. The blue line represents the linear best fit line and R represents the Pearson correlation coefficient.

**Additional file 4. Fig. S3**: Changes in cell ratios across age and race. Area plots showing the average and smoothed cell proportion per compartment across age. Area plots are separated by self-identified race. Line plots showing the cell ratio values stratified by age and self-identified race. Data are represented by the mean values and 95% CIs. The y-axis is the ratio value. The x-axis is age-group and self-identified race is indicated by color, (light-blue = all participants (N = 1295),  dark green = Black women (N = 367), dark purple = White women (N = 895).

**Additional file 5. Fig. S4**: Change in immune cell parameters for paired, longitudinal samples. **A**, **B** Spaghetti plots showing the **A** proportion estimates and **B** absolute counts for paired samples. Points indicate the estimate and either baseline or LLS, and lines connect paired samples. The absolute mean difference and p-values from a paired t-test are below each cell type plot.

## Data Availability

The data generated and/or analyzed during the current study are not publicly available. Authorization from the WHI is needed for access to the data.

## Abbreviations

CBC: Complete blood count
WBC: White blood cell
CMV: Cytomegalovirus
DNAm: DNA methylation
WHI: Women’s Health Initiative
LLS: Long Life Study
DARC: Duffy Antigen Receptor for Chemokines
Neu: Neutrophil
Eos: Eosinophil
Bas: Basophil
Mono: Monocyte
Bmem: B memory
Bnv: B naïve
CD4nv: CD4+ naïve
CD4mem: CD4+ memory
CD8nv: CD8+ naïve
CD8mem: CD8+ memory
Treg: T regulatory
NK: Natural killer
Lymph: Lymphocyte
Tcell: Total T cell
CD4: CD4+ T cell
CD8: CD8+ T cell
Bcell: B cell
Mye: Myeloid
Gran: Granulocyte
NLR: Neutrophil-to-lymphocyte ratio
