# Supplementary figures and images for: Assessment of immune cell profiles among post-menopausal women in the Women’s Health Initiative using DNA methylation-based methods
Source: Clin Epigenetics. 2023 Apr 28;15:69. doi: 10.1186/s13148-023-01488-8 (PMC10141818; doi:10.1186/s13148-023-01488-8)

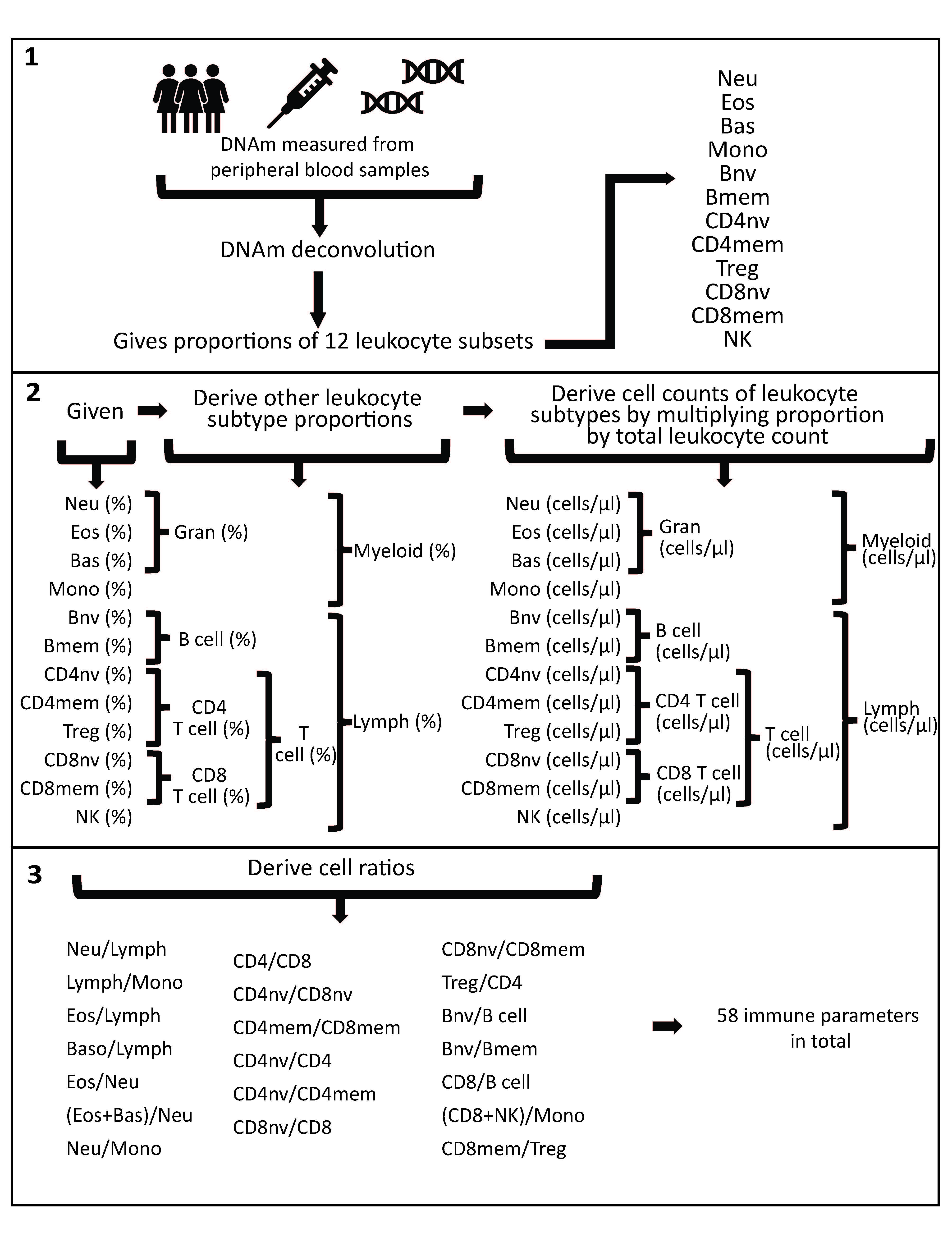

Supplement: Supplementary file 2 — Additional file 2. Fig. S1: Study design for deriving all 58 immune parameters. (1) First, DNAm deconvolution is performed using the DNAm measured from peripheral blood samples. The proportions of 12 leukocyte subsets are given. (2) Derive other leukocyte subsets by adding respective components together. Also, derive absolute counts of all the subsets by multiplying the proportion by the total WBC. (3) Derive cell ratios. This gives 58 immune parameters in total. [file 13148_2023_1488_MOESM2_ESM.jpg]

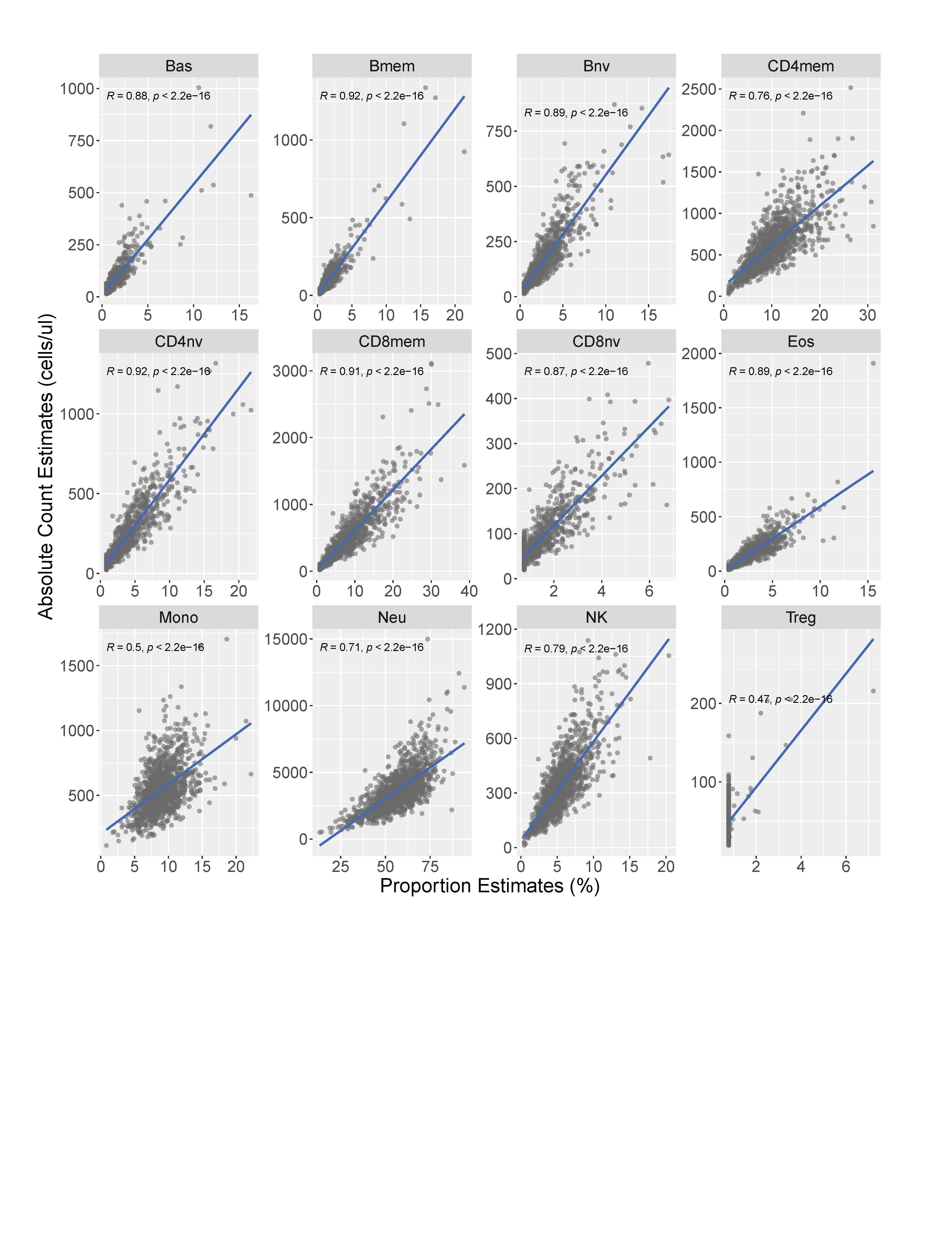

Supplement: Supplementary file 3 — Additional file 3. Fig. S2: Correlation between deconvoluted proportions and derived cell counts. Scatter plots showing the correlation between DNAm deconvoluted proportions and their respective derived cell counts for each of the 12 subtypes. The blue line represents the linear best fit line and R represents the Pearson correlation coefficient. [file 13148_2023_1488_MOESM3_ESM.jpg]

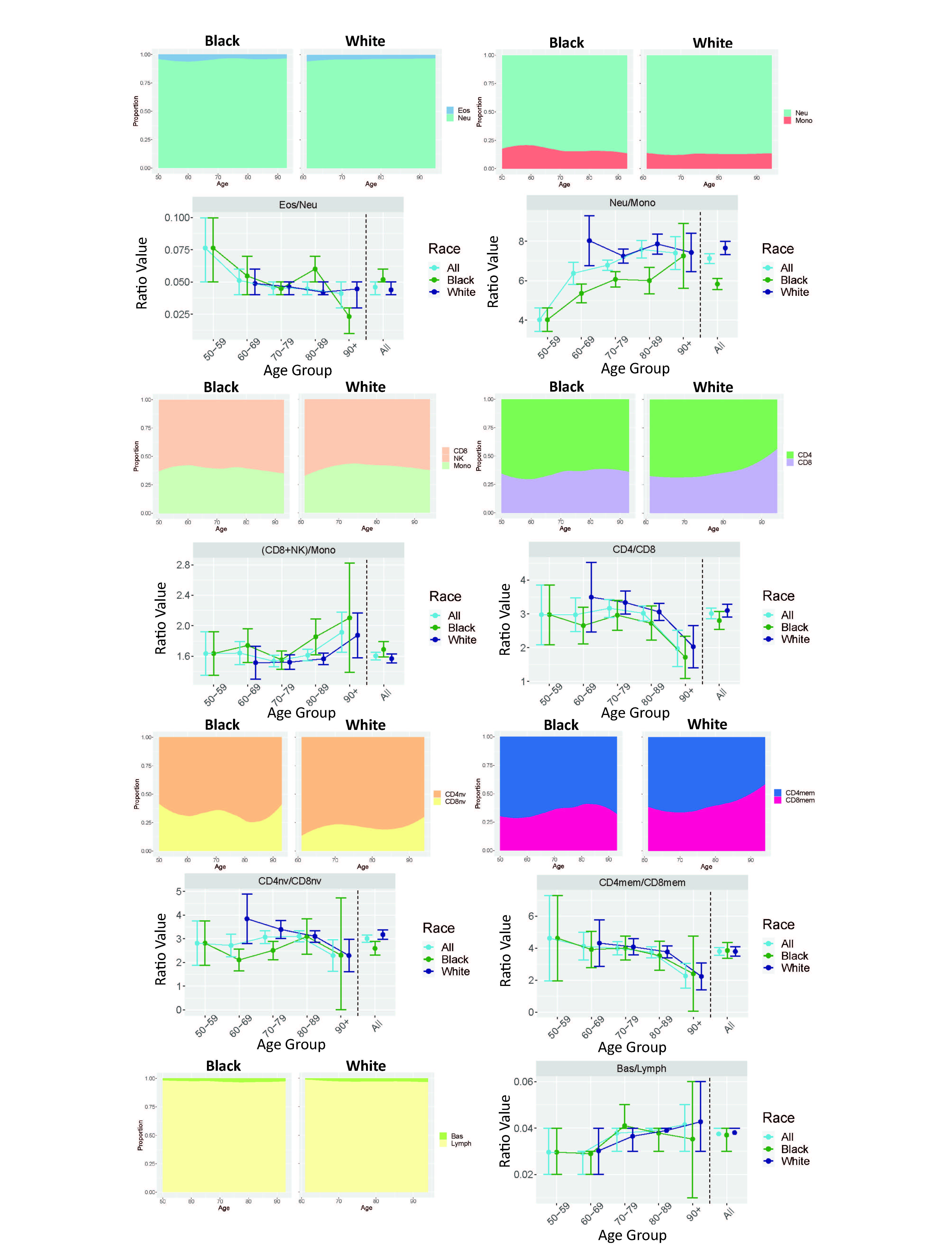

Supplement: Supplementary file 4 — Additional file 4. Fig. S3: Changes in cell ratios across age and race. Area plots showing the average and smoothed cell proportion per compartment across age. Area plots are separated by self-identified race. Line plots showing the cell ratio values stratified by age and self-identified race. Data are represented by the mean values and 95% CIs. The y-axis is the ratio value. The x-axis is age-group and self-identified race is indicated by color, (light-blue = all participants (N = 1295), dark green = Black women (N = 367), dark purple = White women (N = 895). [file 13148_2023_1488_MOESM4_ESM.jpg]

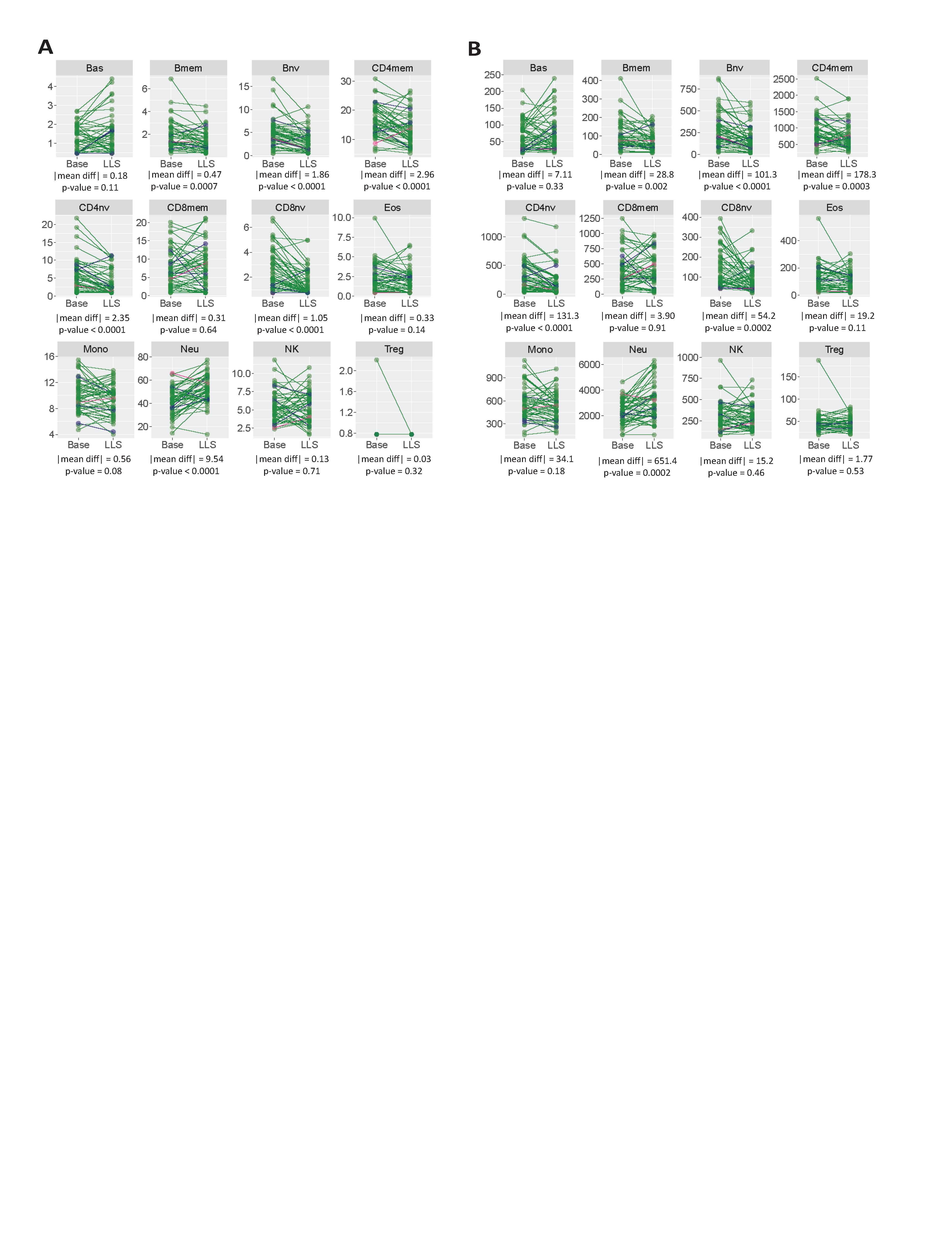

Supplement: Supplementary file 5 — Additional file 5. Fig. S4: Change in immune cell parameters for paired, longitudinal samples. A, B Spaghetti plots showing the A proportion estimates and B absolute counts for paired samples. Points indicate the estimate and either baseline or LLS, and lines connect paired samples. The absolute mean difference and p-values from a paired t-test are below each cell type plot. [file 13148_2023_1488_MOESM5_ESM.jpg]
